# ‘E-cigarette smoking’ is a misleading term: a critical review of its use in academic literature

**DOI:** 10.1007/s11739-025-04014-1

**Published:** 2025-06-18

**Authors:** Yusuff Adebayo Adebisi, Nafisat Dasola Jimoh, Chimwemwe Ngoma

**Affiliations:** 1 College of Social Sciences, University of Glasgow, Glasgow, UK; 2 African Young Leaders for Global Health, Abuja, Nigeria; 3 Knowledge Action Change, London, UK

**Keywords:** Public health communication, E-cigarette, Smoking, Science communication, Public health

## Abstract

The terminology used to describe e-cigarette-related behaviours in academic literature is critical for accurate communication and interpretation. Despite fundamental differences between e-cigarette use (vaping) and tobacco smoking, terms such as “e-cigarette smoking” have been used to refer to vaping, potentially conflating the two behaviours. This study aims to assess the use of the term “e-cigarette smoking” and its variants in peer-reviewed literature and discuss the implications of their usage. A review of academic literature published between 2015 and 2024 was conducted using Embase, Scopus, Web of Science, ScienceDirect, and ProQuest databases. Searches included terms such as “e-cigarette smoking” and related variants, with database-specific filters applied to exclude irrelevant document types. A supplementary search in Google Scholar was performed to capture broader usage, including grey literature. The search identified 613 articles in Embase, 462 in Web of Science, 407 in ScienceDirect, 282 in Scopus and 121 in ProQuest, highlighting the widespread use of “e-cigarette smoking” and related terms. The Google Scholar search returned approximately 4680 results, reflecting the widespread and informal use of “e-cigarette smoking” across diverse publication types, including grey literature. The term has also appeared in highly cited and recent studies in ways that conflate e-cigarette use with smoking, obscuring the fundamental differences between these distinct behaviours. The use of “e-cigarette smoking” and its variants in academic literature has notable implications for research integrity, addiction science, public health, and policy. Misleading terminology can contribute to distorted understandings of nicotine dependence, complicate harm reduction strategies, and risk shaping restrictive policies that fail to differentiate e-cigarette use from smoking. Standardising terminology in academic literature is essential to ensure accurate communication, support evidence-based policymaking, and enhance public health initiatives.

**Supplementary Information:**

The online version contains supplementary material available at 10.1007/s11739-025-04014-1.

## Introduction

Nicotine vaping, or the use of e-cigarettes, also known as electronic nicotine delivery systems (ENDS), has increased significantly over the past decade, alongside the growth of academic literature on the subject [1]. E-cigarettes entered the market around 2007, and by 2021, it was estimated that there were 82 million vapers worldwide [2]. These devices operate by heating a liquid (commonly referred to as e-liquid or vape juice) to produce an aerosol, which is inhaled by users [3]. This process differs fundamentally from smoking, which involves the combustion of tobacco leaves, producing smoke that contains numerous harmful chemical substances and is then inhaled into the lungs [4–6]. Since vaping does not involve combustion, exposure to known tobacco-related toxicants appears to be reduced compared with smoking, making e-cigarettes a potentially lower-risk alternative for nicotine consumption [4].

However, despite these distinctions, terms such as “e-cigarette smoking”, which imply that vaping and smoking are the same, have appeared in academic discourse. Notably, the term “e-cigarette” itself may be problematic, as its inclusion of “cigarette” evokes the imagery and connotations of traditional smoking, potentially making it easier for some researchers, policymakers, and the public to conflate these two distinct behaviours. This misuse of terminology can obscure differences in addiction mechanisms, harm reduction potential, and public perceptions of risk, which are central to addiction science and public health messaging. Such imprecision may not only confuse the interpretation of research findings but also reinforce a linguistic link that undermines the fundamental distinction between aerosolization and combustion. Consequently, the terminology used to describe e-cigarette-related behaviours is an important consideration in peer-reviewed literature, where precision in language is critical [7].

While extensive academic reviews have explored various aspects of e-cigarette research, such as health outcomes and usage patterns [8–10], little attention has been given to the terminology used to describe e-cigarette-related behaviours in the scientific literature. This study aims to review peer-reviewed literature to examine the use of the term “e-cigarette smoking” and its variants and discuss the implications of their usage.

## Method

This study examined the use of the term “e-cigarette smoking” and related variants in peer-reviewed academic literature published between 2015 and 2024. This timeframe was selected to capture a period of substantial growth in e-cigarette research, driven by increased public health attention, regulatory debate, scientific interest, and rising uptake from around 2015 onward. For the purposes of this review, “e-cigarette” refers to electronic nicotine delivery systems that aerosolise e-liquids.

Searches were conducted on 13 December 2024 across five databases: Medline (accessed via Embase), Scopus, Web of Science, ScienceDirect, and ProQuest. Search terms included “e-cigarette smoking,” “electronic cigarette smoking,” “e-cigarette smoker,” “electronic cigarette smoker,” “e-cigarette smoke,” “electronic cigarette smoke,” “e-cigarette smokers,” and “electronic cigarette smokers.” Full search syntax for each database is provided in Supplementary Table 1. Searches were limited to titles, abstracts, and keywords. Filters were applied to exclude references and irrelevant document types such as datasets, patents, theses, and dissertations.

A supplementary search was conducted in Google Scholar to identify additional mentions in grey literature, including reports and working papers. Scopus and Web of Science were selected for trend mapping due to their structured metadata and representative coverage. Other databases were included in the overall citation count but excluded from trend visualisation due to limitations in year-specific export or metadata consistency. In addition to article counts, we extracted and plotted the annual number of citations to these articles within Scopus and Web of Science to assess how the influence of this terminology has evolved over time. Search results were treated independently for each database, and no deduplication was applied across platforms. This was consistent with the descriptive aim of the study, which focused on documenting visibility across indexing platforms rather than estimating a unique publication count.

Terminology misuse was defined as any instance in which the term “e-cigarette smoking” or its variants were used to describe vaping or e-cigarette use. These terms are inherently inaccurate, as e-cigarettes do not involve combustion and are not smoked. Any such usage conflates distinct behaviours and undermines conceptual clarity.

In addition to documenting the presence of these terms across databases, we selected a purposive sample of ten articles for closer examination: the five most-cited articles from Scopus and the five most recent from Web of Science. In Scopus, “most-cited” was based on total citation count as of the search date. Each excerpt containing the term was reviewed descriptively to assess its usage. This interpretative step was informal and did not involve formal coding procedures. Terms were grouped into broad categories such as “behaviour conflation” or “smoker labelling” to support clarity in the results. Mentions that appeared only in reference lists were excluded, as they did not reflect the authors’ own language. Similarly, if “e-cigarette” or a related term appeared at the end of one sentence and “smoking” at the beginning of the next, these were not counted as misuse due to the lack of syntactic or conceptual linkage. All quoted examples are presented exactly as written in the original sources.

## Results

**Fig. 1:**
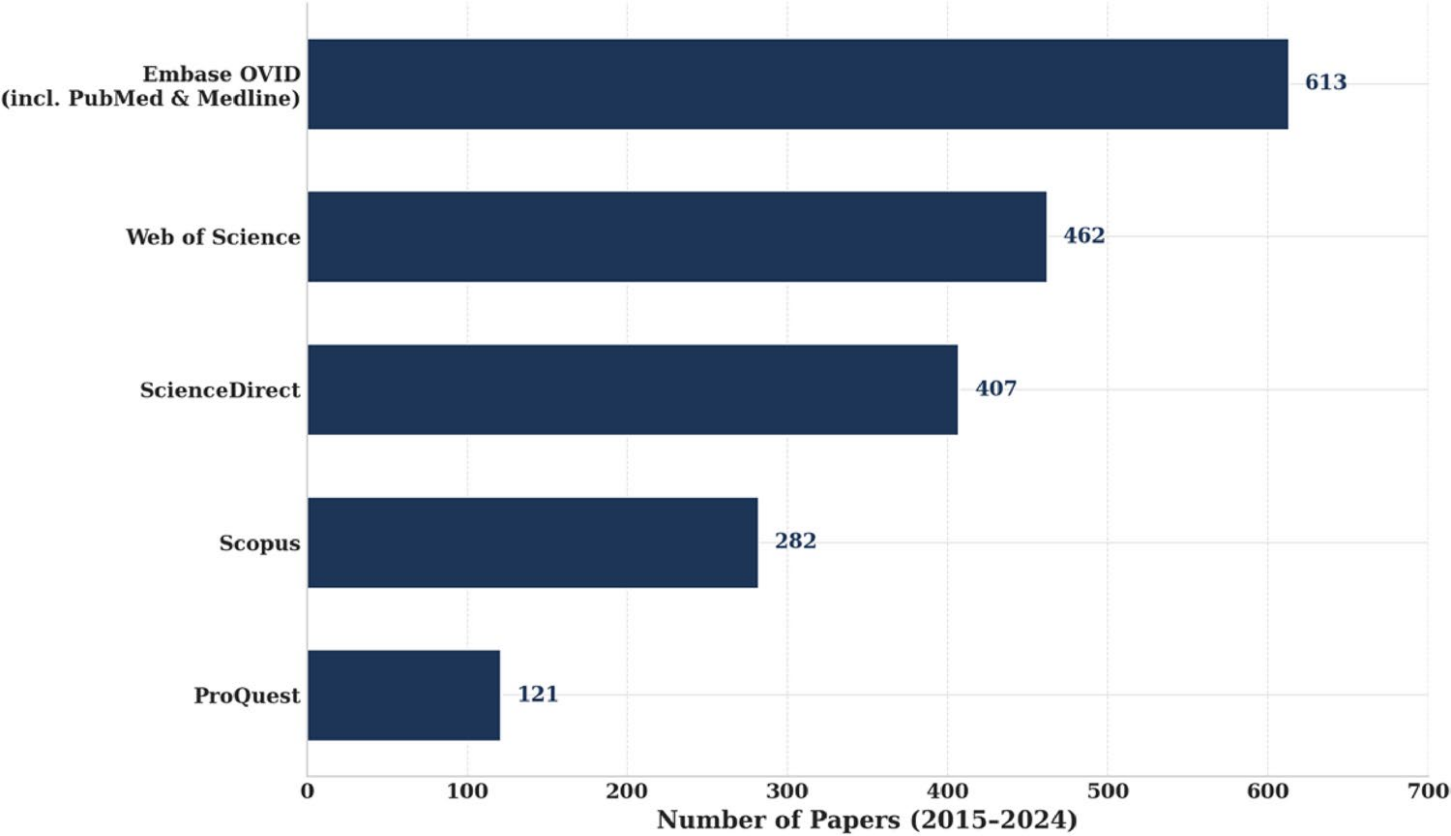
Number of papers containing “e-cigarette smoking” and its variants retrieved from database searches (2015–2024)

The search identified instances of use of the phrase “e-cigarette smoking” and its variants across five databases between 2015 and 2024. Embase (including PubMed/Medline) yielded the highest number of any journal articles (*n* = 613), followed by Web of Science (*n* = 462, any journal articles excluding datasets, patents, dissertations, and grants), ScienceDirect (*n* = 407, original articles and reviews), Scopus (*n* = 282, any journal articles), and ProQuest (*n* = 121, any journal articles) [See Fig. 1]. These figures represent database-specific retrievals, with no deduplication performed across sources. These findings indicate the widespread and entrenched use of “e-cigarette smoking” and related terms in academic literature.

**Fig. 2:**
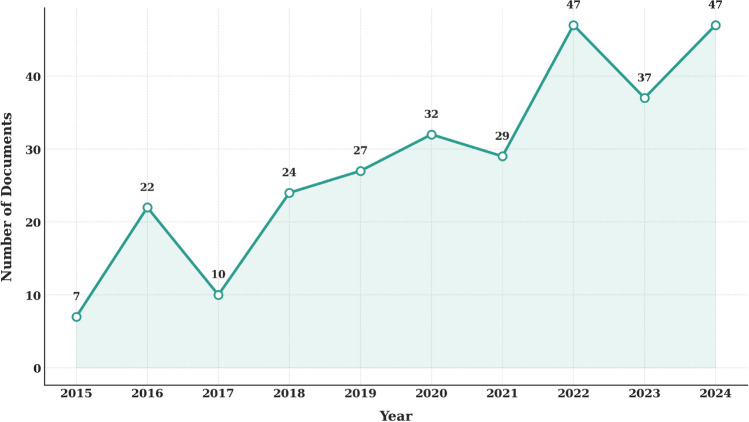
Number of papers in scopus containing “e-cigarette smoking” and its variants (2015–2024)

A supplementary search in Google Scholar, using the same terms and timeframe, returned approximately 4680 results. Its extensive coverage, which includes peer-reviewed articles, theses, conference proceedings, and grey literature, accounts for the higher count (see Supplementary Table 1). The number of Scopus-indexed papers using the term ‘e-cigarette smoking’ or related variants rose overall between 2015 and 2024, starting at 7 papers in 2015 and peaking at 47 in both 2022 and 2024 (see Fig. 2). A notable rise occurred after 2020, with annual counts exceeding 30 from 2021 onward, suggesting the entrenchment of this terminology in recent academic discourse.

**Fig. 3:**
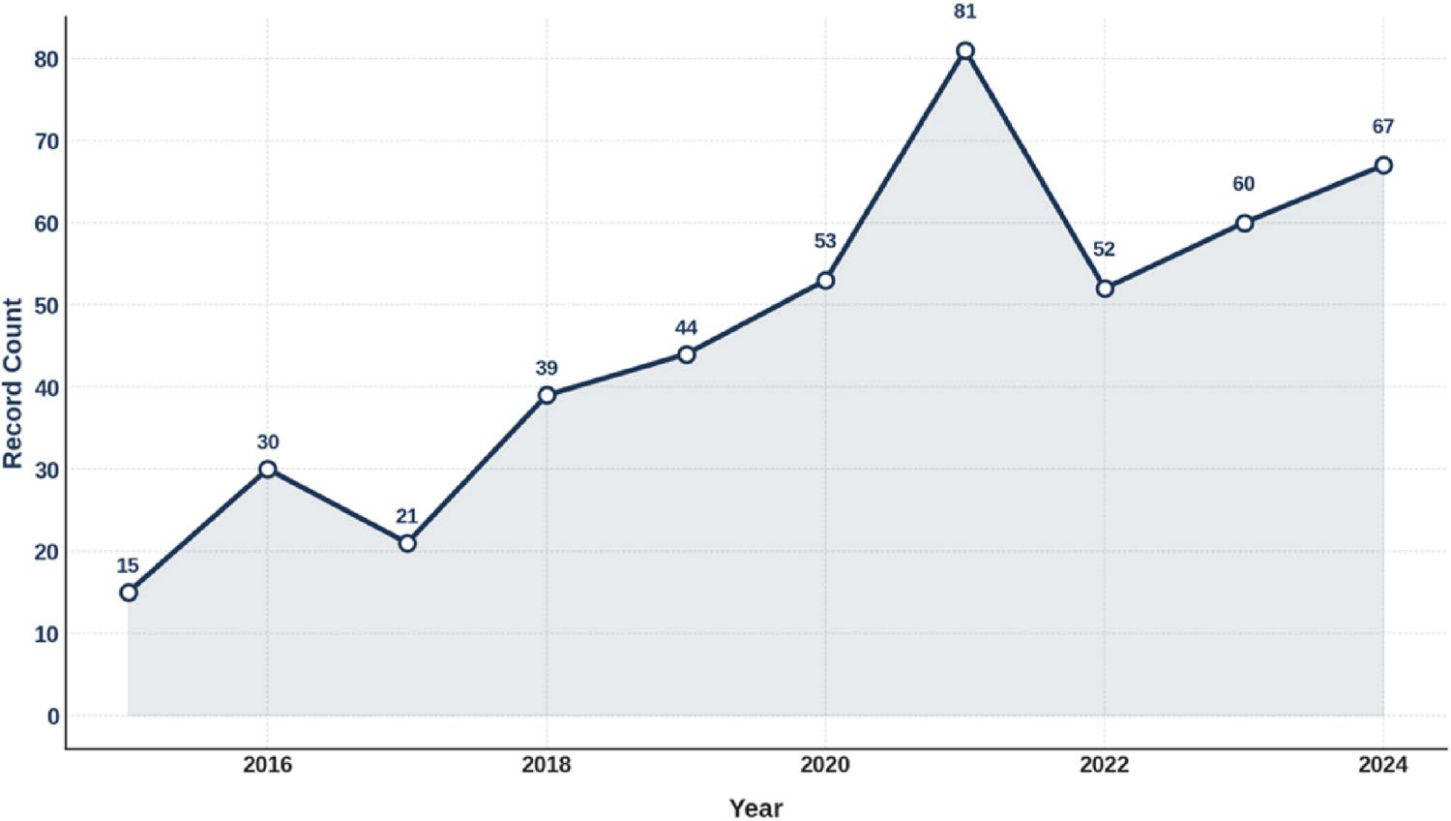
Number of papers in web of science containing “e-cigarette smoking” and its variants (2015–2024)

Figure 3 shows the number of papers in Web of Science containing the phrase “e-cigarette smoking” and its variants between 2015 and 2024. The trend shows a general increase over time, peaking sharply in 2021 with 81 papers. Although there was a dip in 2022, publication numbers rebounded in subsequent years, reaching 67 in 2024.

**Fig. 4:**
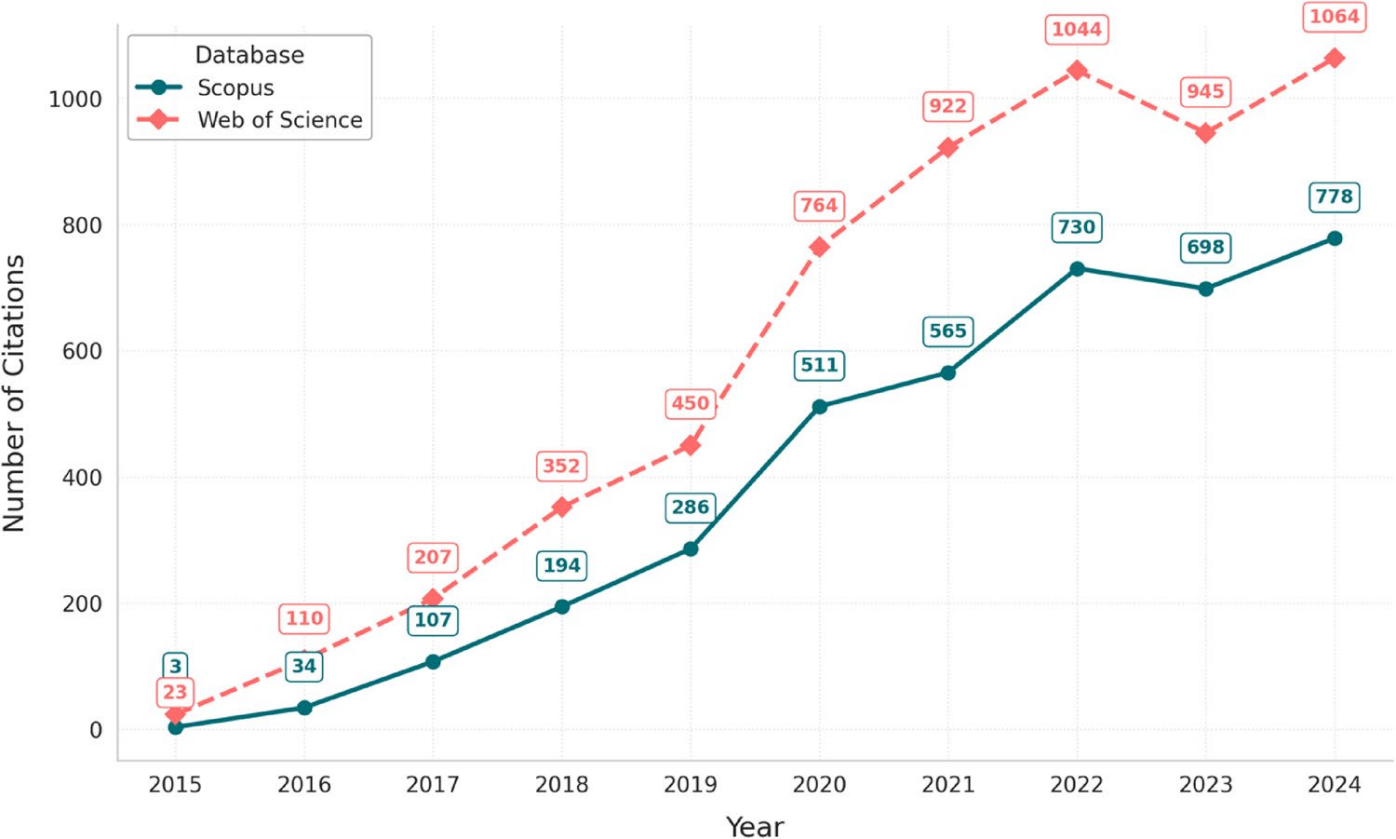
Annual citations of papers using the term “e-cigarette smoking” or its variants in Scopus and Web of Science, 2015–2024

Figure 4 illustrates the annual number of citations for articles using the term “e-cigarette smoking” or its variants in Scopus and Web of Science between 2015 and 2024. Citation counts rose sharply over the ten-year period in both databases, with particularly pronounced growth after 2020. In Scopus, citations increased from just 3 in 2015 to 778 in 2024, totalling 3906 across the decade. Web of Science showed a similar trajectory, starting at 23 citations in 2015 and reaching 1064 in 2024, for a total of 5881. The divergence in magnitude between the two databases was most pronounced from 2020 onward, where Web of Science consistently recorded higher citation counts. These trends reflect both the growing volume and visibility of articles using the term. As some articles may be indexed in both databases, these figures represent citation activity within each platform rather than distinct sets of publications, highlighting the entrenchment of the term in the scientific literature.

Table 1 presents an overview of ten peer-reviewed articles that used the term “e-cigarette smoking” or one of its variants in the context of academic research. The selection includes the top five most-cited articles indexed in Scopus and the five most recent articles indexed in Web of Science between 2015 and 2024. In the Scopus set, all five articles used the term “e-cigarette smoking” when reporting findings related to physiological or health-related outcomes, particularly those involving oxidative stress, endothelial function, or cardiovascular markers. The term appeared in article titles, study descriptions, or comparisons of exposure types. All five instances were categorised under behaviour conflation, where vaping and smoking were described in overlapping or equivalent linguistic terms. In the Web of Science sample, the term was commonly found in cross-sectional research published in 2024. Three articles used smoker labelling, referring to e-cigarette users as “smokers” in demographic classification or participant descriptions. Two articles applied behaviour-conflation or aerosol-as-smoke language, particularly when discussing biological mechanisms or health associations. This review highlights consistent patterns in the appearance of smoking-related language in both older, widely cited literature and recent publications across multiple disciplines.

**Table 1:** Analysis of the top 5 most-cited articles using the term “e-cigarette smoking” and its variants in scopus and the top 5 most recent articles in web of science (2015–2024) [as of December 13, 2024]

**Scopus**				
Ranking based on citation (number of citations)	Journal name (publication year) Study design	Excerpt of some exact sentences with “e-cigarette smoking” and variants	Category	Implications
1 st (286 citations)	Chest (2016) Crossover, single-blind study	Our study showed that *both cigarettes* have unfavourable effects on markers of oxidative stress and FMD after single use, although e-Cigarettes seemed to have a lesser impact “To our knowledge, we provide the first comparison data of the acute impact of traditional tobacco cigarette and *e-Cigarette smoking* on the oxidative stress and on vascular function in smokers and nonsmokers” “Our data suggest that both tobacco and *e-Cigarette smoking* acutely increase oxidative stress and reduce FMD”	Behavior conflation	Normalises the use of “smoking” for vaping and positions both behaviours within the same physiological framework
2nd (218 citations)	European Heart Journal (2020) Narrative review	“Nevertheless, acute *e-cigarette smoking* increases blood pressure, causes endothelial dysfunction and increases vascular and cerebral oxidative stress”	Behavior conflation	Reinforces the perception of similarity between e-cigarettes and cigarettes through repeated use of smoking language
3rd (140 citations)	European Heart Journal (2020) Observational research (involving human subjects) and experimental research (involving animal models and in vitro experiments)	“*Acute e-cigarette smoking* produced a marked impairment of endothelial function in chronic smokers determined by flow-mediated dilation” “With the present study, we found that a single episode of *e-cigarette smoking* induced endothelial dysfunction even in chronic smokers”	Behavior conflation	Embeds vaping in the discourse of tobacco harm without clarifying key exposure differences
4th (138 citations)	Journal of the American College of Cardiology (2016) Randomized crossover experimental design	*Electronic Cigarette Smoking* Increases Aortic Stiffness and Blood Pressure in Young Smokers	Behavior conflation	Uses “smoking” in the title, potentially biasing reader interpretation and media framing despite focus on e-cigarettes
5th (119 citations)	Obstetrical and Gynecological Survey (2018) Review article	“What Are the Differences Between Cigarette Smoking and *E-Cigarette Smoking?*”	Behavior conflation	Treats vaping and smoking as linguistically comparable, possibly confusing distinctions among readers

Web of science				
Study design	Journal name (publication year)	Excerpt of some exact sentences with “e-cigarette smoking” and variants	Category	Implications
Cross-sectional	American Journal of Health promotion (2024)	“The demographic characteristics of the study participants were evaluated by their *e-cigarette smoking* status using chi-squared tests” “Although current evidence does not support a link between *e-cigarette smoking* and colorectal cancer (CRC), the mechanism by which by e-cigarette smoking triggers gut inflammation is similar to the patterns observed with tobacco smoking, which is an established risk factor for CRC”	Behavior conflation; Aerosol‑as‑smoke	Suggests equivalence by using smoking language and linking physiological mechanisms
Cross-sectional	Journal of Egyptian Public Health Association (2024)	“By type, 14%, 12.7%, and 7.3% were cigarette, shisha, and *e-cigarette smokers”* Logistic regression analysis was done to test for predictors of *e-cigarette smoking* among the studied group	Smoker‑labeling	Categorises users as “smokers,” obscuring the behavioural distinction and contributing to definitional confusion
Cross-sectional	Cutaneous and Ocular Toxicology (2024)	“The thickness of the RNFL, GCL, IPL, and choroid was found to be similar in both the healthy *electronic cigarette smokers* and non-smokers groups” “25 healthy *electronic cigarette smokers* and 25 age- and gender-matched healthy non-smokers were included in the study”	Smoker‑labeling	Applies traditional smoking terminology to e-cigarette users, potentially distorting identity and risk classification
Cross-sectional	Discover Public Health (2024)	“In addition, 188 (33.1%) respondents increased *e-cigarette smoking* during the pandemic”	Behavior conflation	Uses “smoking” language in a behavioural survey context, potentially reinforcing misunderstanding in general discourse
Cross-sectional	PeerJ (2024)	“Compared to non-smokers, *electronic cigarette smokers* reported higher levels of severe/extremely severe depression”	Smoker‑labeling	Conflates identity of vapers with that of smokers, which could impact stigma and perceived health risks

## Contextual analysis

As shown in Table 1, the term “e-cigarette smoking” and its variants appear across a range of articles involving diverse study designs, including experimental, observational, and cross-sectional research. This review of selected excerpts highlights how vaping is frequently described using terminology associated with combustible tobacco use. In several cases, vaping and smoking are discussed in parallel when reporting outcomes such as oxidative stress, vascular dysfunction, or endothelial impairment. Although some articles acknowledge differences in magnitude or exposure, the repeated use of the phrase “e-cigarette smoking” aligns vaping linguistically with smoking, potentially framing the two behaviours as physiologically and conceptually similar. Other studies use the term to describe demographic categories or user identity, applying smoker labels to participants who use e-cigarettes even when harm or risk is not discussed. A subset of articles also uses “e-cigarette smoking” in contexts that reference biological mechanisms associated with tobacco exposure, such as inflammation or cardiovascular effects, without clearly differentiating between combustion and aerosolisation. While not all cases involve direct comparisons of risk, the consistent application of smoking-related language to describe nicotine vaping may contribute to blurred distinctions between these behaviours in scientific and public discourse.

The implications of this terminology extend beyond semantics. Referring to e-cigarette use as “smoking” can obscure the fundamental differences in exposure pathways, reinforce inaccurate perceptions of relative harm, and create confusion in communication with policymakers, clinicians, and the public. In clinical settings, such language may influence how patients are classified, how risks are assessed, and how cessation advice is delivered. From a public health perspective, imprecise terminology may hinder harm reduction strategies by discouraging smokers from switching to lower-risk alternatives or by promoting regulatory approaches that treat all nicotine products as equivalent. Even when the underlying findings are valid and robust, the language used to describe vaping can shape how those findings are interpreted. These examples highlight the need for greater precision in scientific communication to support accurate understanding, risk differentiation, and effective policy development.

## Discussion

A fundamental difference between smoking cigarettes and using e-cigarettes centres on the processes of combustion versus aerosolisation [11–13], each carrying distinct behaviours and associated risks. Traditional smoking involves burning tobacco, producing smoke laden with tar, carbon monoxide, and a myriad of toxic, carcinogenic substances [14–16]. These by-products are directly linked to severe health conditions, including lung cancer, cardiovascular disease, and chronic obstructive pulmonary disease [17, 18]. In contrast, e-cigarette use (or vaping) typically entails heating a nicotine-containing liquid (often with propylene glycol, glycerin, and flavourings) to create an aerosol [19, 20]. Because this process does not involve combustion, it largely avoids producing tar, carbon monoxide, and many other harmful chemicals. As a result, while vaping may still expose users to certain substances, these tend to be present at significantly lower concentrations than those found in cigarette smoke [21, 22].

This distinction between combustion and aerosolisation is not merely semantic but chemical and biological. Cigarette combustion occurs at temperatures exceeding 600 °C and produces thousands of harmful by-products, including carbon monoxide (CO), polycyclic aromatic hydrocarbons (PAHs), volatile organic compounds (VOCs), and fine particulate matter [15]. By contrast, e-cigarettes operate at substantially lower temperatures (typically 150–250 °C) and do not ignite or burn any substance [16]. As a result, their aerosol does not contain many of the toxicants associated with combustion. CO, a major biomarker of smoke exposure, is found at negligible levels in e-cigarette users [21], and studies show that switching from cigarettes to e-cigarettes leads to significant reductions in other biomarkers of combustion-related toxicants [22]. For example, levels of NNAL (a metabolite of tobacco-specific nitrosamines), 1-hydroxypyrene (a PAH biomarker), and volatile organic compound metabolites are substantially lower in vapers compared to smokers within days of switching [22]. These findings confirm that e-cigarette aerosol does not carry the same toxicological profile as combustible cigarette smoke.

Beyond their differing toxicological profiles, the sensory and experiential elements of smoking and vaping diverge. Cigarette smoking often brings a harsh, smoke-induced throat hit alongside nicotine delivery [23], whereas vaping provides nicotine in a gentler form, typically with flavoured aerosols and reduced airway irritation [24]. Despite these experiential differences, the visual similarity of inhaling clouds of aerosols and the hand-to-mouth gesture has led to widespread confusion, with vaping frequently mischaracterised as a form of smoking. This conflation is problematic for several reasons. It erases the critical distinction that vaping does not involve burning tobacco, oversimplifies the relative risks of the two behaviours, and confounds both public understanding and policy debates.

There are several possible reasons behind the continued use of terms like “e-cigarette smoking” in the scientific literature. In some cases, researchers may lack awareness of the behavioural and toxicological distinctions between vaping and smoking. In others, familiar language may be used to increase accessibility or align with journal conventions. It is also possible that some authors adopt smoking-related terminology to frame vaping as equally harmful. Editorial policies and legacy indexing practices may further entrench these terms in academic writing. While this study does not attempt to infer intent, recognising these potential influences is critical for designing effective interventions—whether through education, editorial reform, or the adoption of standardised ontologies.

The terminology used in academic literature plays a significant role in shaping public perception and regulatory approaches [7, 25, 26]. The prevalent use of the term “e-cigarette smoking” (and related variants) conflates vaping with smoking, two inherently different activities. Such linguistic inaccuracies have far-reaching implications. First, they obfuscate the essential differences between smoking and vaping. Readers may erroneously equate the health risks of both behaviours, failing to recognise that e-cigarettes, while not harmless, likely pose substantially fewer risks than their combustible counterparts [21, 22]. Second, imprecise language in peer-reviewed literature undermines the credibility and integrity of scientific discourse [27]. Academic publications are held to exacting standards of accuracy. When they adopt misleading terms, there is a heightened risk that policymakers, healthcare providers, and researchers will misinterpret study findings. For instance, a paper examining vaping-related risks that inaccurately labels it as “smoking” could be misread to suggest that vaping carries the same extensive health burdens as traditional cigarette use. Such misinterpretations hamper the development of nuanced harm-reduction strategies and can erode trust in scientific guidance.

These mischaracterisations also have direct implications for clinical practice. Readers, including policymakers or clinicians, might assume the risks are equivalent to those of traditional smoking, which involves far more harmful toxicants [22]. This could exaggerate perceived risks and skew applications of the findings. Physicians may also misclassify patients who vape as smokers, leading to inappropriate application of risk-based guidelines, such as screening for lung cancer using protocols designed for combustible tobacco users. Similarly, during cessation counselling, language conflating vaping with smoking may obscure harm reduction potential, leaving patients uncertain about whether switching offers any benefit. Mislabelling may also distort perceptions of disease risk in conditions like COPD and cardiovascular disease, which are closely tied to long-term combustion exposure. For internists, precision in terminology supports accurate risk stratification, more effective shared decision-making, and clearer communication of evidence-based cessation options. It is important to note that identifying e-cigarettes as non-combustible does not equate to endorsing them as safe but a safer option to combustible cigarette. While some evidence considers them a potentially lower-risk alternative to cigarettes [21], ongoing research continues to assess their long-term impact.

Third, confusing vaping with smoking stigmatises people who use e-cigarette. Over decades, “smoking” has become synonymous with a host of well-documented harms [28]. By using this term to describe vaping, academic literature reinforces negative perceptions that may discourage smokers from exploring e-cigarettes as a reduced-risk alternative. If individuals seeking to quit combustible tobacco perceive vaping as equally harmful, they may remain entrenched in the more dangerous habit, thereby missing potential avenues for harm reduction [29]. This conflation made it more straightforward to apply existing smoking-related laws and restrictions, such as public use bans, advertising curbs, and taxation policies, to e-cigarettes without requiring new legislation. While this alignment may have served regulatory expedience, it also contributed to the spread of harmful misinformation. By promoting the impression that vaping is just as harmful, or even more harmful than combustible cigarette use, such terminology may have deterred adult smokers from switching to a potentially lower-risk alternative. As such, this strategic choice in language may have inadvertently undermined harm reduction goals and deepened stigma against e-cigarette users. Even among specialized researchers, where terminology may seem less consequential, the use of “e-cigarette smoking” can perpetuate inaccuracies in citations, policy briefs, or public communications, amplifying confusion beyond academic circles [31–35].

Fourth, these terminological inaccuracies can misguide public health policies and regulation [30]. If studies fail to differentiate between smoking and vaping, regulations may treat both practices similarly, neglecting the relative reduction in harm that e-cigarettes might offer. Such policy approaches could restrict access to vaping products for adult smokers attempting cessation, inadvertently hindering efforts to reduce smoking prevalence and tobacco-related mortality.

Fifth, the persistent use of “e-cigarette smoking” and its variants risks self-reinforcement. When such terms appear in widely cited research, new authors may adopt them without scrutiny, propagating inaccuracies through subsequent literature. Over time, a cycle of linguistic imprecision takes hold, establishing misleading terminology as normative. Breaking this cycle requires editorial guidelines that discourage conflating vaping with smoking, insist on clarity, and foster the adoption of standard terms (e.g., “vaping” or “e-cigarette use”) that accurately describe these behaviours. This academic misuse is mirrored and amplified in broader discourse, where the term “e-cigarette smoking” has permeated media, policy, and public health narratives. For instance, the BBC reported that “Isle of Man prisoners could be allowed to smoke e-cigarettes in a six-month pilot,” while ABC News noted Los Angeles’ ban on “e-cigarette smoking” in public places, and CNN/Money cited the WHO’s call to prohibit indoor vaping under the same label, framing it as a smoking-like activity [31–33]. NPR quoted researchers warning that “e-cigarette smoking re-normalises cigarette smoking,” and a 2020 Campaign for Tobacco-Free Kids brief argued it “re-glamorises smoking,” both stoking stigma and confusion [34, 35]. Even educational resources, such as the CDC’s PHA STEM lesson plan, refer to “e-cigarette smoking” when teaching about aerosol exposure, further embedding this conflation in public understanding [36]. Similarly, the IARC Advisory Group’s 2025 report notes that chronic exposure (12 weeks) to “e-cigarette smoke (ECS)” induced lung adenocarcinoma in animal models [37]. These examples underline how linguistic inaccuracies extend beyond journals, shaping public understanding, regulatory decisions, and harm reduction efforts.

Sixth, this issue reaffirms the necessity of standardised language in scientific communication. Consistency and clarity not only support effective interdisciplinary collaboration but also enable better translation of research into public health interventions, legislative measures, and informed decision-making [26]. Academic journals can lead this charge by promoting glossaries, standardised keywords, and explicit editorial policies that prioritise precision. For example, Cox et al. [7] have proposed a formal ontology that addresses common terminological confusion in the tobacco and nicotine field. They argue that researchers and policymakers should avoid conflating distinct behaviours and products. Academic journals could adopt these definitions in author guidelines to promote clarity and ontological accuracy. Over time, such standardisation could enhance the precision of research communication, improve comparability across studies, and support clearer risk communication for policymakers, healthcare providers, and the public.

To translate these insights into reform, scientific journals and societies can play a leadership role. Editorial boards, particularly of high-impact journals in public health, clinical medicine, and addiction science, could revise their author instructions to specify the use of precise terminology such as “vaping” or “e-cigarette use”, while discouraging terms that conflate vaping with smoking. Journals aligned with the ICMJE or COPE could develop shared glossaries or checklists that guide authors and reviewers on appropriate terminology. In parallel, scientific societies and guideline-issuing bodies could integrate terminology guidance into position statements and policy documents, ensuring that clinical guidance and public communication accurately reflect the distinct characteristics and risk profiles of nicotine products. Engagement with editorial working groups and journal policy roundtables may offer further opportunities to promote these changes.

In sum, the persistent use of smoking-related terminology in the context of vaping has implications that extend far beyond academic discourse. It shapes public understanding, skews research priorities, and influences global regulatory decisions. Mischaracterising vaping as a form of smoking may discourage harm reduction, distort risk communication, and undermine the development of balanced, evidence-informed policy.

## Limitations

This study is descriptive in nature and does not attempt to assess the impact of terminology misuse on clinical outcomes, patient decision-making, or public health policy directly. While we included contextual analysis of selected excerpts, our review did not conduct formal qualitative coding or evaluate how misused terms shaped the conclusions or recommendations of each study. The search strategy focused on English-language peer-reviewed academic literature and may not capture terminological practices in other languages or informal publications. Furthermore, although we examined a purposive sample of high-impact and recent papers, our analysis was not designed to be exhaustive. We also did not perform deduplication across databases, which may result in some overlap in article counts; however, this was consistent with our aim of mapping visibility across platforms rather than identifying unique publications. Future research could expand on this work by using structured content analysis, evaluating trends across disciplines, or examining how terminology influences citation patterns, guideline development, and patient communication.

## Conclusion

The inaccurate use of the term “e-cigarette smoking” and its variants in academic literature has significant implications for addiction science, public health, and policy. Conflating vaping with smoking undermines efforts to understand the unique mechanisms of nicotine dependence, distorts harm reduction strategies, and risks misinforming public perceptions. Addressing this issue requires a concerted effort to promote accurate, standardized terminology, coupled with rigorous editorial oversight to prevent such conflation. By ensuring clarity and precision in scientific communication, the academic community can better support clear science communication, inform evidence-based policymaking, and strengthen harm reduction approaches. Ultimately, this will provide clearer, more reliable guidance for individuals and stakeholders navigating the complexities of tobacco and nicotine use.

## Supplementary Information

Below is the link to the electronic supplementary material.

Supplementary file1 (DOCX 2732 KB)

## Data Availability

Not applicable.

## Declarations

### Patient and public involvement

Patients and/or the public were not involved in the design, or conduct, or reporting, or dissemination plans of this research.
