# Supplementary material for: ‘E-cigarette smoking’ is a misleading term: a critical review of its use in academic literature
Source: Intern Emerg Med. 2025 Jun 18;20(7):2017–27. doi: 10.1007/s11739-025-04014-1 (PMC12534350; doi:10.1007/s11739-025-04014-1)
Supplement: Supplementary file 1 — Supplementary file1 (DOCX 2732 KB) [file 11739_2025_4014_MOESM1_ESM.docx]

**‘E-Cigarette Smoking’ Is a Misleading Term: A Critical Review of Its Use in Academic Literature**

Yusuff Adebayo Adebisi^1^ Nafisat Dasola Jimoh^2^ Chimwemwe Ngoma^3^

^1^College of Social Sciences, University of Glasgow, Glasgow, UK

^2^African Young Leaders for Global Health, Abuja, Nigeria

^3^Knowledge Action Change, London, UK

Corresponding Author; Yusuff Adebayo Adebisi; Email: [y.adebisi.1@research.gla.ac.uk](mailto:y.adebisi.1@research.gla.ac.uk)

# **Supplementary Table 1: Number of Papers Retrieved from Database Searches (2015–2024)**

| **Database Searched** | **Search Term** | **Total Number of Papers** |
| --- | --- | --- |
| Embase OVID, including PubMed and Medline | ("e-cigarette smoking" or "electronic cigarette smoking" or "e-cigarette smoker" or "electronic cigarette smoker" or "e-cigarette smoke" or "electronic cigarette smoke" or "e-cigarette smokers" or "electronic cigarette smokers").mp. [mp=ti, bt, ab, ot, nm, hw, fx, kf, ox, px, rx, ui, sy, ux, mx, tn, dm, mf, dv, dq]  limit 1 to yr="2015 - 2024" | 613 (Any journal articles) |
| Scopus | TITLE-ABS-KEY ( "e-cigarette smoking" OR "electronic cigarette smoking" OR "e-cigarette smoke" OR "electronic cigarette smoke" ) AND PUBYEAR > 2014 AND PUBYEAR < 2025 | 282 (Any journal articles) |
| Web of Science | TS=("e-cigarette smoking" or "electronic cigarette smoking" or "e-cigarette smoker" or "electronic cigarette smoker" or "e-cigarette smoke" or "electronic cigarette smoke" or "e-cigarette smokers" or "electronic cigarette smokers")  AND PY=(2015-2024)  NOT DT=("Data Set" OR "Awarded Grant" OR "Dissertation Thesis" OR "Patent") | 462 (Any journal articles) |
| ScienceDirect | "e-cigarette smoking" OR "electronic cigarette smoking" OR "e-cigarette smoker" OR "electronic cigarette smoker" OR "e-cigarette smoke" OR "electronic cigarette smoke" OR "e-cigarette smokers" OR "electronic cigarette smokers" | 407 (Original articles and Reviews) |
| ProQuest | (ti("e-cigarette smoking" OR "electronic cigarette smoking" OR "e-cigarette smoker" OR "electronic cigarette smoker" OR "e-cigarette smoke" OR "electronic cigarette smoke" OR "e-cigarette smokers" OR "electronic cigarette smokers")  OR ab("e-cigarette smoking" OR "electronic cigarette smoking" OR "e-cigarette smoker" OR "electronic cigarette smoker" OR "e-cigarette smoke" OR "electronic cigarette smoke" OR "e-cigarette smokers" OR "electronic cigarette smokers")  OR su("e-cigarette smoking" OR "electronic cigarette smoking" OR "e-cigarette smoker" OR "electronic cigarette smoker" OR "e-cigarette smoke" OR "electronic cigarette smoke" OR "e-cigarette smokers" OR "electronic cigarette smokers"))  AND pd(2015-2024) | 121 (Any journal articles) |
| Google Scholar | "e-cigarette smoking" OR "electronic cigarette smoking"  Timeframe: 2015–2024 | ~4,680 (Diverse sources, including peer-reviewed articles, theses, conference proceedings, institutional reports, and preprints) |
